# Signaling Required for Blood Vessel Maintenance: Molecular Basis and Pathological Manifestations

**DOI:** 10.1155/2012/293641

**Published:** 2011-12-06

**Authors:** Masahiro Murakami

**Affiliations:** Yale Cardiovascular Research Center, Section of Cardiovascular Medicine, Department of Internal Medicine, Yale University School of Medicine, New Haven, CT 06511, USA

## Abstract

As our understanding of molecular mechanisms leading to vascular formation increases, vessel maintenance including stabilization of new vessels and prevention of vessel regression began to be considered as an active process that requires specific cellular signaling. While signaling pathways such as VEGF, FGF, and angiopoietin-Tie2 are important for endothelial cell survival and junction stabilization, PDGF and TGF-**β** signaling modify mural cell (vascular smooth muscle cells and pericytes) functions, thus they fortify vessel integrity. Breakdown of these signaling systems results in pathological hyperpermeability and/or genetic vascular abnormalities such as vascular malformations, ultimately progressing to hemorrhage and edema. Hence, blood vessel maintenance is fundamental to controlling vascular homeostasis and tissue functions. This paper discusses signaling pathways essential for vascular maintenance and clinical conditions caused by deterioration of vessel integrity.

## 1. Introduction

The vascular system is not only essential for the maintenance of tissue homeostasis, but also important for managing a wide variety of physiological processes. Blood vessels are actively remodeled and reorganized depending on the tissue oxygen demand, suggesting that the maintenance of blood vessels is an active process achieved by a specific biological program. While the biology leading to new vessel formation has been extensively investigated in the last couple of decades [1], the stabilization of the newly formed vessel and the maintenance of the existing vasculature have not received significant attention. Part of the reason for this under appreciation is the general assumption that blood vessel patency is maintained as a passive process relying on continuous blood flow. This idea has been widely accepted as clinical observations clearly indicate that hemodynamic changes leading to a decrease or cessation of blood flow results in vessel regression [2]. There is little doubt that blood flow is critically important for determining the vessel fate; however, the recent advance of vascular biology strongly argues for the autonomous fate control achieved by blood vessels. Although this is primarily mediated by endothelial cells which appear to have intrinsic mechanisms to sense environmental changes and accordingly remodel blood vessels, recent studies indicate pivotal contribution made by vascular mural cells especially pericytes which can stabilize vessels in part through modulating the endothelial phenotype [3].

The notion of active signaling required to maintain the vasculature is supported by several lines of evidence: (1) as angiogenic growth factors can promote new vessel formation, inhibition of those growth factors in tumor or tissue has been demonstrated to cause vessel regression with variable sensitivity [4–6], (2) disruption of endothelial junctions causes endothelial cell apoptosis leading to disintegration of blood vessels [7, 8], and (3) numerous mouse knockout studies describe vascular insufficiency in the later stage of development at which initial vascular development has been completed and blood circulation is established. These embryos show lethality at E9.5 or later due to vascular fragility leading to uncontrolled hemorrhage and edema. The list of the genes that are implicated in the maintenance of vessel integrity has been provided in the recent review [9], and the number of studies describing the similar phenotype is still growing. In these animals, while blood vessels form initially with normal vasculogenesis and angiogenesis, the maintenance of developing or once-formed vessels is impaired at the later stage of development. This observation implies that vessel formation and maintenance are differently regulated and equally important for producing sustainable tissue perfusion. Furthermore, these studies collectively demonstrated that many cellular and noncellular components in the blood vessel coordinately regulate the maintenance of vessel integrity at varying degrees in different vascular beds. These include endothelial cells, pericytes, smooth muscle cells, fibroblasts, glia cells, inflammatory cells, and the extracellular matrix (ECM) [9].

## 2. Lessons from Clinical Trials for Therapeutic Angiogenesis 

The discoveries of angiogenic growth factors considerably advanced our understanding on biology underlying new vessel formation. This subsequently laid the foundation for new possibilities in the treatment of ischemic diseases over the conventional medical management and invasive procedures such as catheter-based angioplasty and coronary bypass surgery. The new approach to facilitating the endogenous revascularization process has been termed therapeutic angiogenesis. The potential impact of therapeutic angiogenesis in clinical medicine is significant because it will enable us to achieve tissue perfusion by manipulating intrinsic blood vessel growth. However, over the past decades, many attempts to develop biological interventions for therapeutic angiogenesis have not produced significant clinical benefits despite the initial success in preclinical studies. Double-blind randomized clinical trials aiming at biological revascularization in patients with ischemic heart diseases have failed to demonstrate therapeutic efficacy of any single growth factor delivery [10–12]. These studies led to the conclusions that (1) administration of a single growth factor triggering only the initial phase of angiogenesis is not sufficient to drive the whole angiogenic process, (2) vascular maintenance is a crucial process to establish functionally meaningful perfusion, and (3) reevaluation of the detailed biological mechanism of new vessel formation is imperative for the successful clinical application.

As currently understood, new vessel formation is triggered by endothelial cell activation and sprouting coordinated with controlled detachment of the surrounding mural cells, and proteolytic remodeling of the basement membrane and the extracellular matrix. This initial step is followed by assembly into a nascent vascular structure, lumen formation, and, finally, maturation of the newly formed endothelial tube and expression of endothelial cell survival factors. Throughout the process, proangiogenic factors such as vascular endothelial growth factor (VEGF) and fibroblast growth factor (FGF) differentially regulate defined subpopulations of endothelial cells in the angiogenic sprout, promoting endothelial migration at specialized tip cells and proliferation of the stalk cells. Mural cells are then recruited by platelet-derived growth factor (PDGF) and transforming growth factor-*β*1 (TGF-*β*1) to stabilize the nascent blood vessels. In contrast with the initial phase of vessel sprouting, endothelial cells stop migration and proliferation in the maturation step and restore the barrier function of vessels. It is, therefore, reasonably assumed that to counteract the action of proangiogenic factors, vessel stabilization requires activation of distinct cellular signaling pathways different from those that initiate vascular cell mobilization. In fact, VEGF-A, one of the most potent growth factors that initiates strong vascular growth, is known to increase vascular permeability [13], which, in turn, can compromise the barrier function when its action persists. By contrast, other angiogenic growth factors such as fibroblast growth factor 2 (FGF2) and angiopoietin 1 (Angpt-1) do not cause vascular hyperpermeability. While both FGF2 and VEGF-A are potent angiogenic stimulators, capillaries generated by FGF2 are tightly sealed and morphologically different from VEGF-induced capillaries which have multiple fenestra and transendothelial channels [14]. This strongly suggests that VEGF and FGF induce expression of unique subsets of genes by activating distinct signaling programs in the angiogenic process [15]. In view of this, it is considered that understanding of the specific role of each growth factor at specific stages of vascular development is important to successfully control the angiogenic process. Thus, the approach to control vascular stabilization and maintenance needs to be also considered in the practical application of therapeutic angiogenesis in the future.

## 3. Molecular and Cellular Components Important for Vessel Maintenance

### 3.1. VE-Cadherin-Based Endothelial Junction

Endothelial intercellular junctions, critically important for the maintenance of vascular integrity, are comprised of distinct adhesion structures including adherens, tight, and gap junctions [16]. Unlike epithelial junctions which are vertically polarized, endothelial adherens and tight junctions, contributing to the structural maintenance of the endothelium, are intermingled and do not assume stratified localization [17]. Interestingly, in endothelial cells, although VE-cadherin can form a complex with polarity proteins such as Par6 and Par3, this complex lacks atypical-PKC which is required for polarity formation in epithelial cells [18]. This may explain nonstratified endothelial adherens and tight junctions. It is generally regarded that adherens junctions are primarily important for the control of endothelial permeability whereas tight junctions are dependent on the formation of adherent junctions and implicated in blocking the movement of lipids and integral membrane proteins between the apical and basolateral surfaces of the cell [16, 19].

In endothelial cells, VE-cadherin, a calcium-dependent adhesion protein mediating transhomophilic interactions, localizes at cell-cell contacts, regulating the formation of adherens junctions, and linking the site of the junction to the actin cytoskeleton. In the quiescent endothelium, VE-cadherin-based junctions are subjected to continuous reorganization, which renders endothelial junctions highly dynamic and sensitive to extracellular stimuli. In fact, during the process of new vessel formation, endothelial cells undergo dynamic rearrangement upon angiogenic stimuli while continuously reorganizing cell-cell junctions and maintaining barrier function at the same time. This coordination is largely regulated by adhesion molecules at intercellular junctions and is particularly important for tube stabilization and restoration of the full barrier function. Thus, junction proteins play a critical role in controlling vascular integrity both in developing and existing vessels. Among these, VE-cadherin is crucial for this regulation for its capability to remodel actin cytoskeleton via modifying the function of small GTPases [20]. Disruption of VE-cadherin function in the developing vasculature or the adult vasculature results in severe outcomes manifested by significant defects in the vasculature due to vessel collapse, regression, and endothelial apoptosis, leading to extensive hemorrhages [7, 8]. Mouse embryos lacking *Cdh5,* encoding VE-cadherin, die at E9.5 due to defects in vessel remodeling [7].

### 3.2. Pericyte-Endothelial Cell Interaction

Pericytes are mesenchymal-derived cells which are positioned around microvessels and cover gaps between endothelial cells [3, 21]. Although pericytes and endothelial cells are embedded within the same basement membrane, they make special interfaces at peg and socket contacts where the basement membrane is absent and gap and adherens junction constituents are deposited. Besides forming physical contacts, the peg-socket contact is thought to facilitate communication between these two cell types, in which pericytes and endothelial cells respond to signals generated by the counterpart [22]. As has been described extensively in numerous mouse genetic models, failure of pericyte-endothelial cell communication results in severe and often lethal vascular defects at the later stage of embryogenesis exemplified by impaired vascular formation, stabilization, and remodeling. While in these mice, vascular development is initiated by vasculogenesis and angiogenesis, subsequent vessel maturation and stabilization are defective with reduced pericyte coverage. Pericyte-endothelial cell interaction is mediated by multiple growth factor systems including PDGF, TGF-*β*, sphingosine-1-phosphate, Tie-2, and Notch, which are required for pericyte differentiation, recruitment, and expansion. On the other hand, pericyte coverage promotes vessel maturation by resolving angiogenic signals and reducing endothelial proliferation. Alterations in pericyte density or the stable attachment of pericytes to endothelial cells are associated with human pathological conditions such as diabetic microangiopathy, venous malformation, and hereditary stroke and dementia syndrome CADASIL. Moreover, pericytes of tumor vessels present multiple abnormalities, including an abnormal shape and altered gene expression of more immature and less-contractile markers [23]. These pericytes show loose association with the endothelium and extend cellular processes into the stroma. Aberrations in pericyte-endothelial cell communication are attributed to the aggressive nature of tumor angiogenesis and the high incidence of metastasis [24]. Signaling systems and pathological conditions related to pericyte functions are discussed in the following sections.

### 3.3. Extracellular Matrices and Matrix Metalloproteinases

As a major structural component of resistant vessels, the ECM plays a substantial role in the maintenance of vessel integrity. The ECM of the blood vessel wall exists mainly in three forms: the interstitial matrix which fills intercellular spaces of the media and the adventitia, elastic laminae of artery, and the basement membrane which is a sheet-like structure that localizes underneath the endothelium and supports both endothelial cells and pericytes [25, 26]. Reduction of the ECM component in the vascular wall predisposes to compromised vascular structural integrity. For example, patients with osteogenesis imperfecta and Ehlers-Danlos syndrome caused by genetic disorders of major fibrillar collagens such as type I and type III collagen, respectively, often present aortic aneurysm and its rupture in early adult life [27]. Aortic aneurysm also occurs as a result of atherosclerotic changes in which normal architecture of aorta is progressively destructed by increased activity of matrix degrading enzymes, potentially leading to devastating rupture as the disease advances [28]. Besides the degenerated vessel wall, aortic aneurysm features chronic inflammation and a loss of ECM such as medial elastin. Increased matrix metalloproteinase (MMP) production/activity in the vascular wall is thought to be responsible for ECM degradation of this disease. Specifically MMP-2 and MMP-9 have been implicated in the pathogenesis of aneurysm due to their capability to degrade elastin and increased expression levels in the aneurysm tissue and the plasma of patients [29]. Moreover, the imbalance of MMPs and tissue inhibitors of MMPs (TIMPs) is believed to promote disease progression. In the mouse genetic model, whereas either MMP-2 or MMP-9 deficiency is resistant to aneurysm formation [30, 31], disruption of TIMP-1, an MMP-9 inhibitor, leads to exaggerated aneurysm growth [32]. These results confirmed the contribution of MMPs to aneurysm formation and progression through ECM degradation and subsequent impairment of structural integrity of large vessels. 

As ECM degradation by MMPs activates quiescent endothelial cells and thus triggers angiogenesis, constitutive endothelial-matrix interaction is thought to maintain vascular homeostasis and thereby prevent angiogenic vascular growth [33]. Therefore, endothelial attachment to the basement membrane is important for the establishment of stable and mature blood vessels. Besides the basement membrane, the adventitial matrix mainly containing fibrillar collagens and fibronectin produced by fibroblasts also contributes to the maintenance of vascular integrity not only as structural scaffold, but also as a mediator of vascular remodeling in response to flow changes or vascular injury [34]. Recent study revealed the dynamic nature of adventitial fibroblasts which are capable of modulating vascular functions through a paracrine mechanism. Fibroblasts secrete cytokines, growth factors, and reactive oxygen species (ROS), actively controlling endothelial cell interaction with leukocytes and thus the inflammatory process of the vascular wall.

The importance of the ECM in embryonic vascular development has also been shown by an observation of mice lacking histone deacetylase 7 (HDAC7). HDAC7, exclusively expressed in the vascular endothelium in the early embryonic stage, maintains vascular integrity by repressing MMP-10 expression. HDAC7 null mice show embryonic lethality after E11.0 due to a failure of endothelial cell adhesion, leading to vessel dilatation and rupture [35].

### 3.4. Protein Tyrosine Phosphatase and Reactive Oxygen Species (ROS)

Although physiological levels of ROS, serving as a signaling mediator in the vascular system, are important for the maintenance of vascular homeostasis and vascular integrity, oxidative stress induced by elevated levels of ROS may contribute to the initiation and development of vascular dysfunction associated with hypertension and diabetes [36]. The mechanism of action by which ROS cause vascular dysfunction includes protein oxidation by oxidants such as peroxynitrate anion (OONO^−^) and H_2_O_2_. These ROS can increase tyrosine nitration (nY), cysteine (Cys), and zinc thiolate (ZnS_4_) oxidation, affecting important cardiovascular proteins such as eNOS, prostacycline synthase, MnSOD, and sarcoendoplasmic reticulum calcium ATPase (SERCA) [37]. 

Protein oxidation may also lead to junction instability by targeting important components of the junction structure. Increased vascular permeability is usually reversible and does not deteriorate vascular integrity because endothelial cells can quickly restore the barrier function by reestablishing VE-cadherin-based junctions; however, prolonged permeability-inducing stimuli may cause a more profound effect such as accumulation of ROS. ROS can irreversibly inactivate protein tyrosine phosphatases (PTPs) by oxidizing a Cys residue in the catalytic center, thereby affecting the function of PTPs [38]. Given the importance of VE-PTP and other tyrosine phosphatases in the stabilization of the VE-cadherin complex, accumulation of ROS in endothelial cells may impair endothelial barrier function by hyperphosphorylation of VE-cadherin, thus deteriorating vascular homeostasis [39, 40].

## 4. Signaling Systems That Control Vascular Stability

### 4.1. VEGF Signaling

The VEGF gene family consisting of VEGF-A, B, C, D, and placenta growth factor (PlGF) in humans is essential for a wide variety of vascular functions [41]. Multiple isoforms of VEGF generated by alternative splicing differ in their ability to bind heparan sulfate, which determines their bioavailability, and may play distinct roles in vascular development [42]. VEGF-A is a potent angiogenic factor originally identified as a factor capable of increasing vascular permeability through disruption of endothelial junctions [43]. Endothelial junction stability is primarily achieved by VE-cadherin homophilic interactions which form intercellular adherens junctions. VEGF-A induces phosphorylation of Y658 and Y731 at the VE-cadherin cytoplasmic domain via Src activation, disrupting binding of p120-catenin (p120) and *β*-catenin, respectively. Dissociation of catenins especially p120 increases endothelial permeability by dismantling VE-cadherin-based junctions, which is sufficient to maintain endothelial cells in a mesenchymal-like state with a promigratory phenotype [44, 45]. While VEGF controls VE-cadherin stability by inducing catenin uncoupling and VE-cadherin internalization, signaling from cell-cell junctions can also regulate growth factor signaling leading to actin reorganization. In fact, VE-cadherin modulates VEGF-induced Erk1/2 and Akt activation and shear stress response [46, 47]. 

Although VEGF-A disrupts endothelial junctions, which can potentially compromise vascular integrity when the action is prolonged, VEGF signaling is indispensable for physiological endothelial functions and vascular homeostasis. Apart from the angiogenic property, VEGF is widely recognized as a vascular protective factor with its ability to increase nitric oxide (NO) and prostacyclin (PGI_2_) production in endothelial cells [48, 49]. Of these, NO has been implicated in mediating the effects of VEGF on vasodilatation and the maintenance of antiapoptotic signaling through VEGFR2-induced PI3K activation leading to Akt and eNOS phosphorylation. While endothelium-derived NO exerts antioxidant and anti-inflammatory properties, NO produced by both endothelial cells and platelets inhibits platelet aggregation, thereby playing an antithrombotic role. NO has pleiotropic antiatherogenic actions by preventing endothelial dysfunction and smooth muscle cell proliferation which are both required for the initiation and progression of atherosclerosis. Mice lacking eNOS present impaired response to vascular injury and ischemia recovery [50]. 

Endothelial cell activation, critical to triggering an inflammatory response, induces the fusion of Weibel-Palade bodies containing Von Willebrand factor (vWF), tissue plasminogen activator (tPA), P-selectin, IL-8, and endothelin with the plasma membrane. This process transfers these molecules to the cell surface where they promote the recruitment of leukocytes and platelets [51]. NO acts in an anti-inflammatory manner by reducing leukocyte adhesion on the endothelium through inhibiting exocytosis of Weibel-Palade bodies. This process is regulated by the activity of N-ethylmaleimide-sensitive factor (NSF), which is a major component of the exocytotic trafficking machinery [52]. NO appears to inhibit Weibel-Palade body exocytosis from endothelial cells through inhibiting NSF disassembly activity by nitrosylating critical cysteine residues of NSF. 

Moreover, a recent study demonstrated that autocrine VEGF signaling is required for endothelial cell survival in a cell-autonomous manner under nonpathological conditions. Endothelial-specific deletion of the VEGF-A gene in mice leads to progressive endothelial degeneration and sudden death by 25 weeks of age due to vascular insufficiency, suggesting that endogenous VEGF produced by endothelial cells is crucial for vascular homeostasis [53]. Precise regulation of differential VEGF signaling that can promote both endothelial survival and junction disruption is not well understood at this point; however, accumulating evidence points that VEGF coreceptor systems are capable of modifying VEGF signaling outcomes in a context-dependent manner. Several studies described an essential role of VE-cadherin in VEGF-induced Akt activation required for endothelial cell survival [7, 54]. VE-cadherin, sensing cellular spatial information depending on cell density, modulates VEGF signaling by controlling VEGFR2 endocytosis that is required for Erk1/2 activation [46, 55]. Thus, endothelial cells are able to differently respond to VEGF stimuli in order to accommodate the environment and tissue demands.

 The family of Roundabout (Robo) transmembrane receptors is the canonical receptor for the signaling molecule Slit, regulating commissural axon guidance [56]. Robo4 is an endothelial cell-specific member of the Robo receptor family, which is another signaling system that has the capacity to modulate VEGF signaling. Robo4 inhibits VEGF-induced vascular permeability and angiogenesis by binding and signaling through UNC5B. Thus, Robo4-UNC5B signaling in endothelial cells plays a role in the maintenance of vascular integrity by antagonizing the action of VEGF [57].

Observations of tumor vasculature provided insightful information with regard to mechanisms of vessel fragility and leakiness, which is potentially applicable to anticancer therapy [58]. VEGF is recognized as a key factor required for the growth of tumors with its inherent overproduction in many human malignancies [59]. The tumor microenvironment is characterized by hypoxia, low pH, and high interstitial fluid pressure, all of which underlie exaggerated production of proangiogenic factors such as VEGF through HIF1*α* activation in the tumor milieu. Unregulated VEGF expression causes vascular hyperpermeability that leads to leakage of plasma proteins into the stroma, further stimulating persistent angiogenesis and creating a self-reinforcing vicious cycle [60]. As a result, the abnormal tumor vasculature showing chaotic networks composed of heterogeneous immature vessels is formed. Structurally, in these tumor vessels, endothelial cells have wide junctions and multiple fenestra with loose association with pericytes and the basement membrane, resulting in hemorrhage and edema which limit efficient perfusion. Therefore, normalization of tumor vessels using anti-VEGF strategies has emerged as a new approach to cancer therapy [58, 61]. VEGF inhibition prunes the immature vasculature including excess branches and enhances pericyte coverage, stabilizing vessels and decreasing tumor vessel permeability. These anti-VEGF effects lead to improvement of tumor perfusion and oxygenation, resulting in increased sensitivity to chemotherapeutic agents [62, 63].

### 4.2. FGF Signaling

FGFs comprise one of the most versatile and complex signaling families in vertebrates, playing critical roles in a wide variety of biological processes [64]. FGFs are broad-spectrum mitogens that stimulate various cellular functions including migration, proliferation, and differentiation [65]. FGF2 is recognized as a cell survival factor that inhibits apoptosis in many cell types including endothelial cells [66]. The expression pattern of FGFs is very variable, ranging from nearly ubiquitous (FGF1 and 2) to highly restricted to particular cell subsets at specific developmental stages (FGF3, 4, 8, 17, and 19). In pathological conditions such as angiogenesis, inflammation, and malignancies, FGFs are abundantly secreted from various cell types including monocytes, tissue macrophages, endothelial cells, stroma cells, and tumor cells [65].

Despite the recognition of FGF as a strong in vivo angiogenic promoter, deciphering its precise functions in the vascular system has suffered from the lack of information obtained from mouse knockout studies [67, 68]. Disruption of *Fgfr1* or *Fgfr2* in mouse embryos results in embryonic lethality at very early stages of development, precluding further evaluation of their contribution to vascular development [64]. On the other hand, knockout studies of angiogenic FGF ligands such as FGF1 and FGF2 failed to show abnormalities in embryonic vascular development, implying that extensive redundancy exists in the ligand system [69]. One of the difficulties investigating the FGF system arises from its promiscuous actions to a variety of cell types and tissues; however, using tissue-specific promoters, recent studies began to uncover roles played by FGFs in the cardiovascular system. While endothelial FGF signaling is dispensable for mouse coronary vascular development, myocardial FGF signaling appears to be essential for triggering hedgehog activation that is required for VEGF expression and coronary vessel formation [70]. Furthermore, analyses of FGF signaling in the adult vasculature revealed significant contribution of FGF to vascular development as well as vascular integrity maintenance. In the adult mice, basal endothelial FGF signaling is required for vascular homeostasis. Inhibition of FGF signaling leads to disassembly of endothelial junctions, progressing to severe deterioration of vascular integrity [71]. In clear contrast to VEGF which induces disruption of VE-cadherin-based junctions by Src activation, FGF fortifies junction adhesiveness via enhancing coupling of VE-cadherin with p120 catenin. The critical role of FGF signaling to new vessel formation is also demonstrated in a more recent study in which the mechanism of signaling crosstalk between FGF and VEGF is described. VEGFR2 expression levels are tightly controlled by endothelial FGF signaling capable of upregulating VEGFR2 transcription via an Ets-dependent manner; thereby FGF promotes neovascularization indirectly by regulating endothelial responsiveness to VEGF [72]. It has been repeatedly shown that while FGF-induced new vessel formation is often disrupted by VEGF inhibition in various in vivo angiogenic models, VEGF-induced vascular formation is not as much affected by FGF signaling depletion [67]. Together, these studies suggest the hierarchic control of new vessel formation by which the FGF system promotes new vessel growth via controlling VEGF signaling.

### 4.3. Angiopoietin-1 and Tie2

The angiopoietin- (Angpt-)Tie2 signaling system has crucial roles in vascular functions including angiogenesis and vessel maintenance [73]. Angpt-1 is a ligand of the endothelial cell receptor Tie2, and activation of Tie2 signaling enhances endothelial cell barrier integrity and endothelial-pericyte interaction, thereby promoting vascular stabilization [74]. While expression of Tie2 is largely specific to the endothelium, Angpt-1 production by mural and perivascular cells facilitates basal Tie2 signaling in quiescent endothelial cells that, in turn, is required for endothelial homeostasis. In contrast, Angpt-2, produced and stored in Weibel-Palade bodies in endothelial cells, normally functions as an Angpt-1 antagonist. Angpt-2 destabilizes the quiescent vasculature and activates endothelial cells to respond to angiogenic stimuli. Overexpression of Angpt-2 in the mouse endothelium attenuates physiological Tie2 signaling and thus increases vascular permeability, suggesting that Angpt-2 inhibits Tie2 signaling and counteract the Angpt-1 action [75]. 

Mice lacking Angpt-1 or Tie2 have similar cardiovascular defects, indicating the importance of the Angpt-1-Tie2 signaling system in cardiovascular development. These mice die mid gestation (E10.5–E12.5) due to the absence of hierarchical vascular development and perturbed vascular integrity, manifested by reduced pericyte coverage and detachment of pericytes from the endothelium [73]. Although Tie2 signaling is thought to be indispensable for vascular development as previous studies indicated, a recent study using the tissue-specific gene deletion strategy in mice, however, reached the conclusion suggesting that it may not be the case [76]. Detailed analysis of Angpt-1^−/−^ embryos showed that the earliest detectable defect seen at E9.5 is loss of myocardial trabeculations while the vascular system appears normal. Thereafter, these embryos become markedly growth retarded with generalized disorganization of blood vessels. When Angpt-1 is specifically excised from cardiomyocytes using the Nkx-2.5 Cre-driver line, the vascular defects reported in the global knockout are recapitulated, indicating that vascular phenotypes are dependent on the cardiac defect and resulting from impaired blood circulation. Moreover, deletion of Angpt-1 in the later stage of vascular development does not affect pericyte number or vascular mural cell recruitment. Together, authors concluded that Angpt-1 is not required for embryonic vascular development or maintenance of vascular quiescence; however, it functions as a protective factor, modulating responses to tissue injury and microvascular abnormality in diabetes [76].

Recent studies began to reveal molecular mechanisms of Angpt-1 modulating vascular functions. Angpt-1 inhibits VEGF-induced Src activation through RhoA activation which leads to Src association with mDia (a RhoA downstream target) and sequestration of Src from VEGFR2 [77]. Moreover, Angpt-1 induces translocation of Tie2 to cell-cell contacts and bridges Tie2 proteins, resulting in the formation of the transdimer of Tie2. Although functional contribution of this Tie2 homophilic interaction to junctional stability and permeability control is unclear, Angpt-1 preferably transmits PI3K-Akt signaling in quiescent cells in the presence of Tie2 transdimers with their close association with eNOS. In contrast, in isolated endothelial cells, Erk1/2 signaling which promotes cell migration and proliferation is activated by Angpt-1 [78, 79].

### 4.4. Sphingosine-1-Phosphate

Sphingosine-1-phosphate (S1P), a sphingolipid metabolite found in high concentrations in platelets and blood, is a lipid mediator that interacts with GPCRs (S1P_1_–S1P_5_) to induce a variety of biological responses [80, 81]. S1P is an endothelial survival factor capable of producing NO through Akt activation [82]. It is also able to enhance endothelial barrier integrity through S1P_1_ receptor (Edg1) signaling by promoting Rac1 activation and adherens junction assembly [83]. Administration of the S1P receptor agonist FTY720 in vivo potently blocks VEGF-induced vascular permeability, suggesting that S1P receptor on endothelial cells is able to regulate vascular permeability [84]. Furthermore, the S1P_1_ receptor is essential for normal vascular function since systemic antagonism of S1P_1_ receptor under basal physiological conditions causes S1P_1_ receptor internalization and degradation through receptor phosphorylation, leading to enhanced pulmonary vascular leakage [85, 86]. 

Genetic studies further indicate the importance of S1P in the vascular system. S1P_1_ knockout mice die between E12.5 and E14.5 due to severe hemorrhage resulting from a defect in the vascular stabilization process [87]. In mice in which S1P_1_ is specifically deleted from endothelial cells, the phenotype mimics the one obtained from the embryos globally deficient in S1P_1_ whereas vascular smooth muscle cell-specific knockout has no effect [88]. While these data demonstrate that S1P_1_ signaling in the endothelium is critical for the regulation of vascular maturation, the lack of vascular maturation in these animals is also attributed to impaired endothelial-pericyte interaction mediated by N-cadherin. Although structural and functional basis of endothelial-pericyte interaction has not been well characterized, N-cadherin is reported to localize at the interface of these two cell types in the embryonic brain vasculature [89]. This N-cadherin-based junction mediates pericyte adhesion to endothelial cells, thereby contributing to vessel maturation and stabilization [90]. S1P stimulation of endothelial cells results in activation of Rac1, promoting forward trafficking of N-cadherin to the plasma membrane and the formation of the N-cadherin-catenin complex [90]. Inhibition of N-cadherin profoundly attenuates the process of vascular stabilization in vitro and in vivo, suggesting the specific contribution of S1P signaling to N-cadherin-induced pericyte attachment [91].

### 4.5. PDGF Signaling

The role of platelet-derived growth factor (PDGF) signaling in the vascular system is established as an important player to mediate pericyte-endothelial interaction [92]. PDGFs are major mitogens for mesoderm-derived cells such as fibroblasts, pericytes, and smooth muscle cells, and for some cell populations of neuroectodermal origin [93]. In the mouse embryo, perivascular mesenchymal cells expressing PDGFR*β* respond to PDGF-BB (a PDGF-B homodimer) produced by the angiogenic endothelium. Paracrine PDGF signaling is thus required for mural cell recruitment and expansion as has been demonstrated that PDGF-B expression is particularly abundant in tip cells of angiogenic vessels and in the endothelium of growing arteries [94]. The significant contribution of PDGF signaling promoting mural cell investment to vascular barrier integrity has been shown by mouse genetic studies. Both PDGF-B and PDGFR*β* null mutant mice die perinatally, displaying lethal hemorrhage and edema caused by a pericyte loss in microvessels [95]. The results of endothelium-specific knockout of PDGF-B further confirmed that PDGF-BB generated from angiogenic endothelial cells is critically important for recruitment and proliferation of mural cell progenitors in vicinity [96]. The absence of pericytes in capillaries increases their diameters and generates microaneurysms by endothelial hyperplasia, suggesting that the pericyte coverage negatively control endothelial cell proliferation. The detailed analysis of PDGFR*β*-deficient mice revealed that vessel instability observed in these mice is modified by systemic upregulation of VEGF. The endothelial junction structure is slightly altered in PDGFR*β*-deficient mice, and this is attributed to the VEGF effect, since the onset of endothelial hyperplasia precedes the endothelial junction abnormality [97]. 

It appears that initial induction of pericyte differentiation from mesenchymal progenitors is independent of PDGF signaling and is most likely mediated by other factors such as TGF-*β*. Pericyte populations in different tissues are affected in varying degrees by the loss of PDGF signaling in developing PDGFR*β* ^−/−^ embryos. Therefore, PDGF signaling is thought to be important in the subsequent maturation process in the angiogenic vessels where PDGF-BB released from endothelial cells drives pericyte migration and expansion [94]. This endothelium-pericyte interplay is particularly crucial for the formation of the blood-brain barrier (BBB), a specific physical barrier of the brain capillaries. Using pericyte-deficient mouse mutants which have defective PDGF signaling in the embryonic or adult vasculature, recent studies clearly showed that pericytes are necessary for the formation of the BBB, and that absolute pericyte coverage determines the extent of vascular permeability. Interestingly, in the central nervous system vasculature, the formation of tight junctions and endothelial vesicle transport by transcytosis are the critical regulator of vascular permeability which is increased by pericyte deficiency [98, 99].

### 4.6. TGF-*β* Signaling

TGF-*β* family includes TGF-*β*s, activins, inhibins, nodal, bone morphogenetic proteins (BMP), and growth differentiation factors (GDF), comprising one of the largest growth factor/cytokine families in vertebrates. Although TGF-*β* family members are known to play crucial roles in the vascular development, their precise role especially in endothelial functions is still controversial [100]. This is probably attributable to the remarkable diversity of their actions and complex regulation of the signaling system. In fact, TGF-*β* effects are highly context-dependent with in vitro observations suggesting that TGF-*β* is stimulatory to endothelial cell functions at low concentrations whereas it can be inhibitory at high concentrations [100]. Moreover, the presence of pericytes in the endothelial cell culture leads to TGF-*β* activation, which, in turn, inhibits endothelial proliferation and migration through VEGFR2 downregulation [101, 102]. Therefore, one of the important roles of TGF-*β* signaling in mural cells is to attenuate the endothelial cell response to angiogenic stimuli, thereby limiting vessel overgrowth and resolving the active angiogenic process. This appears to form a negative feedback loop by which endothelial cells promotes pericyte differentiation and expansion that lead to vessel stabilization and, consequently, vessel maturation.

Genetic studies in mice have indicated the essential role of TGF-*β* in pericyte functions and vascular development. Disruption of the TGF-*β*1 gene in mice leads to extraembryonic vascular defects exemplified by the failure of endothelial differentiation accompanied with fragile yolk sac vasculature [103]. Inactivation of *Tgfbr2* in the smooth muscle cell lineage results in vascular defects in the yolk sac and embryonic lethality between E12.5 and E16.5, suggesting impairment of mural cell recruitment [104]. The importance of TGF-*β* signaling in mural cell function is further endorsed by the observation that mice with neural crest-specific deletion of *Tgfbr2* develop a phenotype similar to that of DiGeorge syndrome due to the failure of neural crest derivative differentiation into smooth muscle cells in the cardiac outflow tract [105]. Moreover, mouse phenotypes resulting from targeted disruption of TGF-*β*RII, endoglin or activin receptor-like kinase 1(ALK1) are highly reminiscent of TGF-*β*1 null mice, showing vascular abnormalities characterized by systemic vascular dysplasia and recurrent hemorrhage caused by telangiectases and arteriovenous malformations [100]. These studies collectively demonstrate the importance of TGF family genes in the various aspects of vascular development.

### 4.7. Notch Signaling

The Notch signaling pathway is an evolutionarily conserved, intercellular signaling system that plays important roles in a wide variety of biological processes. During vascular development, Notch signaling plays essential roles in endothelial cell specification including angioblast specification, arteriovenous differentiation, and tip/stalk cell formation [106, 107]. The critical contribution of Notch signaling to the endothelium and new vessel formation has been well characterized in mouse and zebrafish studies. Genetic ablation of Notch1 or a Notch ligand, Delta-like ligand 4 (Dll4) causes vascular deformation and embryonic lethality due to defective arterial and venous specification of endothelial cells [108, 109]. In this process, Notch is a downstream of VEGF signaling, inducing arterial differentiation through activation of PLC*γ*, MAPK, and EphrinB2/EphB4 signaling. EphrinB2, which is an arterial endothelial marker, is a direct transcriptional target of Notch signaling [110]. As signaling components of the Notch pathway are critically involved in arteriovenous specification, deregulation of Notch signaling causes aberrant direct communication between an artery and a vein, leading to arteriovenous malformation (AVM). In mouse and zebrafish genetic models, both loss and gain of function mutations can result in the formation of arteriovenous malformation and embryonic vascular remodeling defects [106]. 

When Dll4 binds Notch1, the intracellular domain of Notch1 (NICD) is cleaved by *γ*-secretase and translocates to the nucleus where it regulates gene transcription. In the vascular sprout of the retina, while the VEGF-A gradient generated by astrocytes in the ischemic area induces endothelial cells at the leading edge to adopt a tip cell phenotype, Dll4 prominently expressed in the tip cell signals to following stalk cells through Notch1 to downregulate VEGFR2 and inhibit filopodia formation [111, 112]. Thus, during vascular patterning, Notch signaling is required for stalk cell specification by actively suppressing the tip cell phenotype, controlling the number of tip cells and vascular sprouts. In fact, ectopic activation of Notch signaling in the mouse retina reduces tip cell filopodia and the vascular density [113].

Notch signaling appears to be required for vessel stabilization and homeostasis through multiple mechanisms. Notch signaling inhibits endothelial cell proliferation and motility, thus preventing excessive vessel sprouting and stabilizing newly formed vessels. Although increased Notch signaling does not necessarily induce vessel instability, recent study revealed that coordination of Notch and Wnt signaling plays a role in stabilization of nascent vessels. Notch-regulated ankyrin repeat protein (Nrarp), directly induced by Notch signaling, promotes canonical Wnt signaling and controls the stability of newly formed vessel anastomosis [114]. Notch signaling in vascular smooth muscle cells also contributes to arterial specification/differentiation and thus to vessel stabilization. Genetic disruption of Notch3, expressed in smooth muscle cells of arteries, leads to arterial defects including enlargement of arteries with thin smooth muscle layers [115]. Interestingly, endothelium-specific knockout of Jagged1 causes defects in smooth muscle cell differentiation, whereas endothelial Notch signaling and arteriovenous differentiation occur normally. This suggests that endothelial-mural cell crosstalk mediated by Notch signaling is necessary for proper arterial development. Endothelial Jagged1 promotes the development of neighboring vascular smooth muscle through Notch3 activation, and the loss of Jagged1 in the endothelium causes a deficiency of smooth muscle cell recruitment and differentiation, thus leading to vascular instability [116, 117].

## 5. Clinical Manifestations of Impaired Vessel Maintenance

The semipermeable compartmentalization achieved by blood vessels especially by the endothelium is critically important for normal organ functions and tissue homeostasis [118]. The proximal disorders resulting from the breakdown of the barrier function are bleeding and tissue edema. The impairment of the barrier integrity can result from prolonged stimuli of permeability-inducing agents such as VEGF or genetic abnormalities that lead to fragile vessels causing by unstable endothelial junctions, impaired vessel specification/stratification, and pericyte insufficiency. These congenital vascular abnormalities are collectively referred to as vascular malformation which includes several different types according to the type of the blood vessel predominantly affected. Among them, venous malformations are the most common form. Impaired vessel specification leads to an aberrant direct communication of an artery and a vein, which is termed as arteriovenous malformation (AVM). The majority of vascular malformations is sporadic and has no genetic components; however, recent studies identified a variety of genetic abnormalities which provide clues to pathogenesis of vessel abnormalities and mechanisms of vascular maintenance [119–122].

### 5.1. Cutaneomucosal Venous Malformations

Venous malformations, the most common type of vascular malformation, comprise either superficial or deep veins that are abnormally formed and dilated in the skin, mucous membrane, or in any organ system characterized by lesions composed of enlarged, tortuous venous channels [121]. Although venous malformations are usually present at birth, due to the slow nature of disease progression, they may not be diagnosed at young ages. Genetic abnormalities contribute only 2% of reported venous malformations; however, recent studies revealed mutations in the TEK gene, encoding Tie2, in the families with dominant inheritance of venous malformations [123]. The mutations identified thus far have been located at the cytoplasmic domain of Tie2, resulting in ligand-independent phosphorylation and activation of the receptor. These autosomal-dominant venous malformations, termed venous malformations, multiple cutaneous, and mucosal (VMCM), tend to involve small multifocal, bluish mucocutaneous lesions. In addition to the inherited form of VMCM, somatic mutations causing loss of function of Tie2 is suggested to have a role in the etiology of solitary or multiple sporadic venous malformations, which are the more common form of the disease [124].

### 5.2. Hereditary Hemorrhagic Telangiectasia (HHT)

Hereditary hemorrhagic telangiectasia (HHT), also known as Osler-Weber-Rendu syndrome, is a genetic disorder that is inherited as an autosomal dominant trait and affects 1-2 in 10,000 individuals [120, 125]. HHT causes abnormal blood vessel formation in the skin, mucous membranes, and often in organs such as the gastrointestinal tract, lungs, liver, and brain [120]. The lesions are characterized by mucocutaneous telangiectases, and in some cases, life-threatening visceral arteriovenous malformations. Recent genetic studies identified responsible genes associated with HHT, all of which are components of TGF-*β* family signaling pathways. HHT type I results from mutations in *ENG*, coding for endoglin, a coreceptor of TGF-*β* receptors. HHT type II causes by mutations in *ACVRL1* which encodes ALK1, a type I receptor for the TGF-*β* superfamily ligands. While mutations of either of the two genes account for most clinical cases, a small subset of patients carry mutations in *MADH4* encoding Smad4, a transcription factor that mediates TGF-*β* signaling. 

Mouse phenotypes resulting from targeted disruption of components of TGF-*β* signaling pathway such as TGF-*β*1, TGF-*β*RII, endoglin, or ALK1 are all leading to severe vascular abnormality and embryonic lethality at mid gestation, in some part recapitulating HHT phenotypes [100]. 

Detailed analysis of HHT gene functions using mouse models of tissue-specific or haploinsufficient gene inactivation has begun to reveal the mechanism of HHT development [120, 126]. Current understanding of HHT pathogenesis indicates that HHT results from endoglin or ALK-1 haploinsufficiency, where the remaining wild-type allele is unable to express sufficient protein for normal function. HHT mutations are thought to modify predominantly endothelial cell responses to TGF family ligands mediated by endoglin and Alk1 since they are relatively endothelial specific genes. However, because the vast majority of blood vessels in HHT patients appear to develop and function normally, perturbation of a pathological process that requires finely-tuned TGF-*β* signaling such as wound healing and angiogenesis might play as an extra trigger. While endothelial ALK1 is essential for the establishment of proper arteriovenous (AV) connections during vascular development, in the adult endothelium lacking ALK1, AVM forms following activation of the quiescent endothelium by wound that elicits angiogenesis [127]. In HHT, the augmented angiogenic response including excessive endothelial proliferation and exaggerated vessel sprouting is partially attributable to impaired recruitment of mural cells to the newly formed vessel due to reduced endothelial cell secretion of TGF-*β*1 or reduced TGF-*β*1-induced responses. In HHT type I patients, circulating levels of TGF-*β*1 are reduced [128]. Moreover, paracrine TGF-*β* signaling is defective in mice with endothelial-specific deletion of TGF-*β*RII or ALK5 as evidenced by reduced phosphorylation of Smad2 in the adjacent mesothelial cell layer of the yolk sac. Phosphorylation of Smad2 and differentiation of smooth muscle cells can be rescued by exogenous TGF-*β*1 in the yolk sac culture, consistent with the idea that lower levels of TGF-*β* or reduced responsiveness required for receptor activation play a role in HHT pathogenesis [129]. 

In this regard, the recent discovery of thalidomide as a potential therapeutic agent for HHT confirmed the importance of mural cell dysfunction responsible for the formation of fragile vessels. In mice heterozygous for a null mutation in the endoglin gene, thalidomide treatment stimulates mural cell recruitment by increasing PDGF-B expression in endothelial cells and thus rescues vessel defects [130].

### 5.3. Cerebral Cavernous Malformations (CCMs)

Cerebral cavernous malformations (CCMs) are sporadic or inherited vascular malformations in the central nervous system characterized by dilated, thin-walled capillary-like channels without intervening brain parenchyma. They are one of the commonest vascular malformations in the brain, affecting roughly 0.1–0.5% of the general population [131]. Genetic studies have identified autosomal dominant mutations associated with CCMs: *KRIT1* (CCM1), *CCM2* (MGC4607, Malcavernin, OSM), and *PDCD10* (CCM3) [132]. Although neither of these CCM proteins are structurally related to each other nor have been implicated as angiogenesis inducers, recent studies indicated possible mechanisms leading to vascular malformation. KRIT1, originally identified as a Rap1a interacting protein, is partially localized at cell-cell contacts and the loss of KRIT1 accounts for the unstable endothelial junctions. It has been shown that CCM proteins exist as a large protein complex, and a defect of one protein can affect the function of other CCM proteins [133]. KRIT1 or CCM2 is capable of inhibiting RhoA and ROCK which can disassemble endothelial junctions and cause hemorrhage [134]. Since Rap1 is able to enhance junction stability and KRIT1 is a Rap1 effector, there is a possibility that the loss of KRIT1 directly affects endothelial junctions in a Rap1-dependent manner. Rap1 regulates the junctional localization of KRIT1, which is required for Rap1-mediated endothelial junction stabilization [135]. 

Recent mouse genetic studies advanced our understanding of pathogenesis leading to CCM. Disruption of heart of glass (HEG1), a transmembrane receptor previously implicated in CCM functions in zebrafish, results in defective integrity of the heart and the vasculature in mice. HEG1 coupled with CCM proteins through KRIT1 is required for vascular development and endothelial junction formation [136]. Haploinsufficiency of *Ccm2* in mice, a genotype equivalent to that in human CCM, results in impaired endothelial barrier function. Interestingly, loss of CCM2 leads to activation of RhoA, and the impaired barrier function in heterozygous mice is restored by simvastatin, a drug known to inhibit Rho GTPases [137]. Moreover mice with global or endothelial cell-specific inactivation of *Ccm3* show defects in embryonic angiogenesis and die at an early embryonic stage. In response to VEGF stimulation, CCM3 is recruited to and stabilizes VEGFR2, thereby playing a pivotal role in VEGFR2 signaling [138].

### 5.4. Capillary Malformation-Arteriovenous Malformation (CM-AVM)

Capillary malformation-arteriovenous malformation (CM-AVM) is a recently discovered hereditary disorder characterized with atypical cutaneous multifocal capillary malformations often in association with high-flow lesions such as arteriovenous malformations of the cerebrospinal and visceral organs or arteriovenous fistulas. Genetic studies identified loss of function mutations in RASA1, which encodes p120-RasGAP [121, 139]. As GTPase activity leads to, by hydrolysis of GTP to GDP, inactivation of small GTPases, loss of p120-RasGAP function suggests the possibility to increased Ras and downstream MAPK activation. Furthermore, p120-RasGAP has been shown to interact with p190-RhoGAP, which inhibits RhoA through a p120 catenin-dependent mechanism and is required for adherens junction formation [140, 141].

### 5.5. Cerebral Autosomal-Dominant Arteriopathy with Subcortical Infarcts and Leucoencephalopathy (CADASIL)

The clinical relevance of Delta-Notch signaling is manifested by the cerebral small vessel disease: cerebral autosomal-dominant arteriopathy with subcortical infarcts and leukoencephalopathy (CADASIL), now recognized as the most common cause of inherited stroke and vascular cognitive impairment in adults [106, 142]. CADASIL is inherited as an autosomal dominant trait, resulting from mutations in *NOTCH3*, which causes degeneration of smooth muscle cells of cerebral small vessels and accumulation of the Notch extracellular domain (NECD) at the surface of residual smooth muscle cells. These changes lead to thickening of the vessel wall and a reduction of cerebral blood flow [142, 143]. Genetic studies revealed that among 33 exons, all CADASIL mutations occur in exon 2–24 of the Notch3 gene within 34 EGF-like repeats, leading to an odd number of cysteine residues [142].

Genetic disruption of *Notch3* in mice causes structural defects of small arteries because of impaired differentiation and maturation of arterial smooth-muscle cells; however, total loss of Notch3 does not cause CADASIL pathology [115]. Furthermore, CADASIL-causing mutations of Notch3 can activate RBP-J*κ* transcription comparable to wild-type levels and do not seem to affect Notch signaling per se [144, 145]. Therefore, although the precise cause of the disease is still unclear, recent studies suggest that gain of novel function of the mutant protein, possibly arising from novel protein-protein interactions rather than defective Notch3 function, is a likely mechanism for the CADASIL pathogenesis.

As discussed earlier, Notch pathway is important for arteriovenous differentiation and vessel patterning during embryonic vascular development, and deficiency of Notch signaling can cause arteriovenous malformations [146, 147]. Expression of constitutively active Notch4 (int3) in the mouse endothelium develops features of brain arteriovenous malformations characterized by cerebral arteriovenous shunting and vessel enlargement [148, 149]. Conditional activation of the Notch1 gene in endothelial cells of mouse embryos also causes defects in vascular remodeling progressing to arteriovenous malformations [150]. Analysis of brain arteriovenous malformations revealed that Notch1 signaling is upregulated in smooth muscle and endothelial cells of the lesions surgically resected from human patients [151].

## 6. Conclusion and Perspective

**Figure 1:**
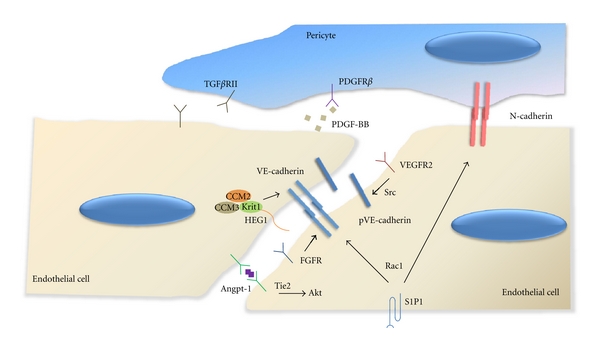
Signaling pathways controlling vascular maintenance. While VEGF signaling disrupts VE-cadherin-based junctions through Src-mediated VE-cadherin phosphorylation and internalization, FGF signaling promotes p120 association with VE-cadherin, thus increasing VE-cadherin stability at adherens junctions. Angpt-1 binding to Tie2 at cell-cell contacts leads to formation of Tie2 transdimers which activates Akt and promotes cell survival. S1P binding to S1P1 (Edg1) is able to stabilize endothelial junctions via Rac1 and promotes N-cadherin forward trafficking required for endothelial-pericyte interaction. HEG1-CCM signaling at endothelial junctions enhances junctional stability through Krit1 interaction with *β*-catenin in the VE-cadherin complex. PDGF-BB secreted from endothelial cells recruit pericytes expressing PDGFR*β*. TGF-*β* produced in endothelial cells induces mural cell differentiation. TGF*β*RII is also expressed in endothelial cells and controls various endothelial functions.

Regulation of the vascular barrier function is crucial for tissue homeostasis. Recently, our understanding of molecular mechanisms leading to vessel stabilization has significantly expanded owing to the advance of basic research followed by critical appraisal of therapeutic angiogenesis trials. It has also become apparent that the maintenance of existing vessels requires active cellular signaling which share common features with signaling mechanisms involved in the vessel maturation process of new vessel formation. The signaling systems controlling vascular maintenance is summarized in Figure 1. Components of signaling pathways involved in vascular stability often cause vascular malformations as disease genes. Conversely, genes identified through genetic studies of vascular diseases most likely play a role in physiological regulation of vascular integrity. As has been discussed, vessel maintenance is successfully achieved by orchestrated actions of growth factors and cytokines that are capable of modifying the function of vascular cells especially endothelial cells and mural cells. Although indispensability of these cell types and signaling pathways required for the maintenance of vessel integrity have been unequivocally demonstrated by numerous studies, it is important to further elucidate detailed molecular mechanisms of signaling interactions between different cell types in the vasculature. The endothelial-pericyte junctions are anatomically identified; however, signals exchanged during new vessel formation and vessel maintenance between these cells are not clearly understood. Besides genetic components leading of vascular instability, epigenetic factors are also playing an important role in modifying disease manifestations such as the location and the severity of the vascular abnormality in the presence of ubiquitous, germline mutations. Further investigations exploring this aspect including the “two-hit” mechanism for disease development and presentation should provide significant insights into our understanding to vascular maintenance. 

As regulation of vessel maintenance is a fundamental vascular function associated with a wide variety of vascular disorders and disease conditions, elucidating the precise mechanisms will benefit the development of new approaches for therapeutic angiogenesis and vascular malformations as well as cancer treatment.
